# The impact of altering emission data precision on compression efficiency and accuracy of simulations of the community multiscale air quality model

**DOI:** 10.5194/gmd-16-1179-2023

**Published:** 2023-02-20

**Authors:** Michael S. Walters, David C. Wong

**Affiliations:** 1 Atmospheric and Environmental Systems Modeling Division, Center for Environmental Measurement and Modeling, Office of Research and Development, US Environmental Protection Agency, Research Triangle Park, NC, USA; 2 Oak Ridge Associated Universities, Oak Ridge, TN, USA

## Abstract

The Community Multiscale Air Quality (CMAQ) model has been a vital tool for air quality research and management at the United States Environmental Protection Agency (US EPA) and at government environmental agencies and academic institutions worldwide. The CMAQ model requires a significant amount of disk space to store and archive input and output files. For example, an annual simulation over the contiguous United States (CONUS) with horizontal grid-cell spacing of 12 km requires 2–3 TB of input data and can produce anywhere from 7–45 TB of output data, depending on modeling configuration and desired post-processing of the output (e.g., for evaluations or graphics). After a simulation is complete, model data are archived for several years, or even decades, to ensure the replicability of conducted research. As a result, careful disk space management is essential to optimize resources and ensure the uninterrupted progress of ongoing research and applications requiring large-scale, air quality modeling. Proper disk-space management may include applying optimal data-compression techniques that are executed on input and output files for all CMAQ simulations. There are several (not limited to) such utilities that compress files using lossless compression, such as GNU Gzip (gzip) and Basic Leucine Zipper Domain (bzip2). A new approach is proposed in this study that reduces the precision of the emission input for air quality modeling to reduce storage requirements (after a lossless compression utility is applied) and accelerate runtime. The new approach is tested using CMAQ simulations and post-processed CMAQ output to examine the impact on the performance of the air quality model. In total, four simulations were conducted, and nine cases were post-processed from direct simulation output to determine disk-space efficiency, runtime efficiency, and model (predictive) accuracy. Three simulations were run with emission input containing only five, four, or three significant digits. To enhance the analysis of disk-space efficiency, the output from the altered precision emission CMAQ simulations were additionally post-processed to contain five, four, or three significant digits. The fourth, and final, simulation was run using the full precision emission files with no alteration. Thus, in total, 13 gridded products (4 simulations and 9 altered precision output cases) were analyzed in this study.

Results demonstrate that the altered precision emission files reduced the disk-space footprint by 6 %, 25 %, and 48% compared to the unaltered emission files when using the bzip2 compression utility for files containing five, four, or three significant digits, respectively. Similarly, the altered output files reduced the required disk space by 19 %, 47 %, and 69% compared to the unaltered CMAQ output files when using the bzip2 compression utility for files containing five, four, or three significant digits, respectively. For both compressed datasets, bzip2 performed better than gzip, in terms of compression size, by 5 %–27 % for emission data and 15 %–28 % for CMAQ output for files containing five, four, or three significant digits. Additionally, CMAQ runtime was reduced by 2 %–7 % for simulations using emission files with reduced precision data in a non-dedicated environment. Finally, the model-estimated pollutant concentrations from the four simulations were compared to observed data from the US EPA Air Quality System (AQS) and the Ammonia Monitoring Network (AMoN). Model performance statistics were impacted negligibly. In summary, by reducing the precision of CMAQ emission data to five, four, or three significant digits, the simulation runtime in a non-dedicated environment was slightly reduced, disk-space usage was substantially reduced, and model accuracy remained relatively unchanged compared to the base CMAQ simulation, which suggests that the precision of the emission data could be reduced to more efficiently use computing resources while minimizing the impact on CMAQ simulations.

## 1 Introduction

The Community Multiscale Air Quality (CMAQ) model (Byun and Schere, 2006) is a sophisticated, 3D Eulerian (gridded) numerical modeling system based on message passing interface (MPI) that uses scientific first principles to simulate the chemical transformation and transport of ozone, particulate matter, toxic compounds, and acid deposition. Since the formation and transformation of chemical species are functions of complex atmospheric and chemical interactions, two primary input types are required to initialize CMAQ simulations: meteorology and emissions. First, meteorological data (such as temperature, wind, cloud formation, and precipitation rate) provide atmospheric conditions to drive CMAQ. The second required input field, which is the focal point of this study, is emission data (i.e., emission rates from emission sources) that characterize pollutants from both man-made and naturally occurring sources.

The CMAQ model typically requires multiple emission datasets which occupy a significant amount of disk space. Although disk space is becoming progressively cheaper and more affordable, the research and computational needs are rapidly increasing and becoming more complex. For instance, the total sizes of emission and meteorological datasets are about 7.0 and 6.8 GB, respectively, for a 1 d CMAQ simulation for the contiguous United States (CONUS) with a horizontal resolution of 12 km. The total disk-space size for 1 d of output is 20GB (for a typical output configuration considering only surface output and neglecting extra diagnostic output). Including 3D fields and diagnostic output, however, the total output disk-space size can easily be tripled. Most studies with CMAQ on this scale create at least a full year’s worth of data, so aggressive disk-space management is justifiable to minimize overall costs associated with running CMAQ. Aggressive disk-space management could be a substantial cost-saving measure, regardless of whether simulations are conducted on-site (such as with a high-performance computing architecture or a Linux cluster) or by using cloud computing, where data retrievals can quickly elevate costs. Here, we propose optimizing disk space by compressing CMAQ emission datasets as one practical consideration to maximize storage capacity. If successful, this option could be extended to other input types with large disk-space needs, such as meteorological data.

Compression algorithms can be described as either lossless or lossy. Lossless compression algorithms reduce disk space by replacing repeated sequences with a smaller, unique identifier. Thus, an entire dataset can be retrieved, once uncompressed, without alteration of the original dataset (hence the name, lossless). Lossy algorithms, however, in terms of numeric arrays, reduce disk space by manipulating the mantissa of individual floating-point numbers. Typically, trailing, or insignificant bits, are replaced with a sequence of zeros or ones. As a result, data are compressed at the cost of numerical inconsistencies between the original dataset and the compressed dataset.

The concept of maximizing disk space by altering netCDF datasets has been examined previously by Zender (2016) and Kouznetsov (2021). Zender (2016) created a versatile toolset that compresses data based on user specifications that are applied to the mantissa of floating-point datasets. The first notable algorithm developed by Zender (2016) is precision trimming, which is publicly available in the netCDF operators (NCOs, http://nco.sourceforge.net/nco.html, last access: 11 April 2022) utility. Precision trimming sets all non-significant bits to zero (bit shaving) which, based on analysis, produces an undesirable bias of the compressed data (Zender, 2016). As a result, Zender (2016) introduced a Bit Grooming algorithm (default algorithm in the NCO) that shaves (to zero) and sets (to one) the least significant bits of consecutive values. Despite the additional toolset, Kouznetsov (2021) found substantial artifacts, or numerical inconsistencies, in multipoint statistics caused by Bit Grooming. Due to the suboptimal results, Kouznetsov (2021) developed and evaluated multiple lossy compression algorithms with respect to the NCO’s available toolsets from Zender (2016). Kouznetsov (2021) created a round and half-shaved lossy compression algorithm which both doubled compression accuracy by rounding the mantissa to the nearest value that has zero tail bits and by setting all tail bits to zero, except for the most significant bit which gets set to one (Kouznetsov, 2021).

Excluding analyses conducted on datasets via lossy compression algorithms, the authors are unaware of any studies that have been conducted on the compression efficiency of floating-point datasets with respect to *n* significant digits. Additionally, Zender (2016) and Kouznetsov (2021) did not conduct evaluations regarding the impact of altered precision datasets on numerical simulations. In this study, the precision of netCDF datasets will be reduced and compressed to explore compression efficiency, and the resultant reduced precision datasets will be used to run CMAQ simulations to quantify the impacts on runtime and on model accuracy as a result of dataset manipulation via a lossy compression algorithm. This study proceeds as follows: in Sect. 2, a description of the methodology will be provided, followed by results in Sect. 3 and then the conclusions in Sect. 4.

## 2 Methodology

All input and output files in this study are 32-bit, binary, netCDF files which inherently contain seven or eight significant digits at most. To perform this study, we created a simple tool written in Fortran to truncate floating-point data in netCDF files by keeping *n* significant digits which are normalized in scientific notation. Table 1 shows several examples of this numerical manipulation. We applied this tool to alter the precision of two different datasets (input emission and CMAQ model output) by keeping *n* significant digits.

**Figure 1.**
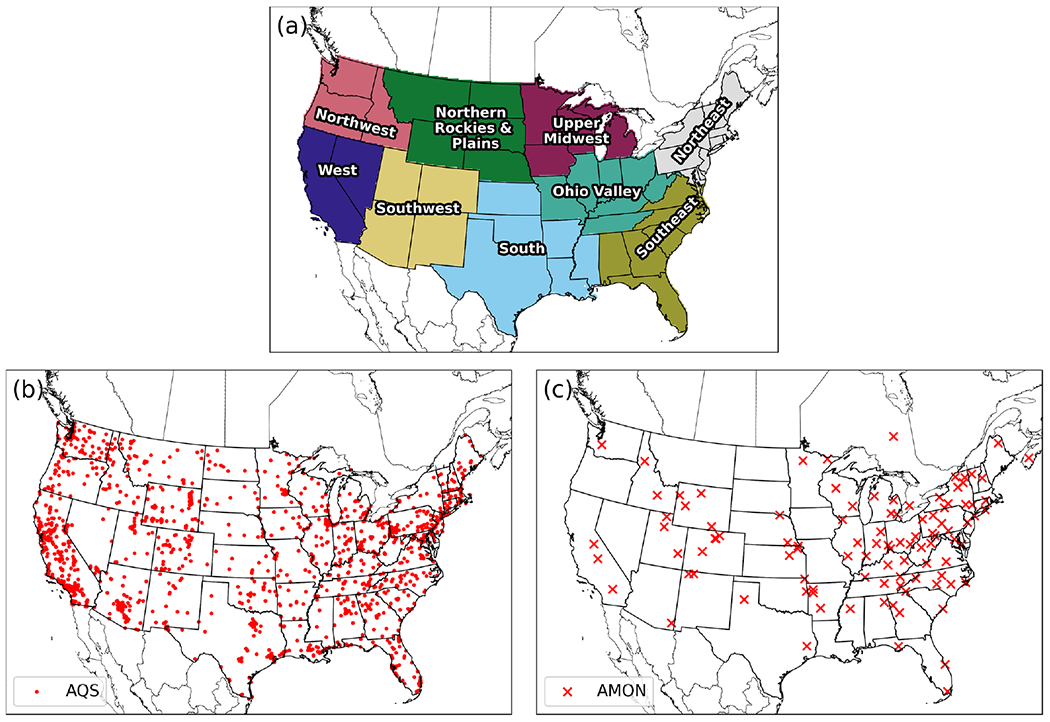
Regions for spatial and temporal stratification (**a**), AQS stations (**b**), and AMON stations (**c**) for the proceeding evaluation.

For this study, CMAQ v5.3.1 (USEPA, 2019; Appel et al., 2021) was run with 459 columns, 299 rows, and 35 vertical layers with a horizontal grid-scale resolution of 12 km (Fig. 1a). Emission input files consist of two area sources and nine point sources (hourly). The area-source emission files contain 57 and 62 variables, and the point-source files contain anywhere from 54 to 58 variables (containing one vertical layer). Ten CMAQ output files (nine of them are hourly) were generated in this study: three output files were generated for simulation-restart purposes (SOILOUT, CGRID which contains only 1 h data, and MEDIA), two files contained average (APMDIAG and ACONC) and hourly (CONC) species concentrations, three files held wet deposition (WETDEP1; 140 variables), dry deposition (DRYDEP; 174 variables), and deposition velocity (DEPV; 104 variables) output, and lastly, the final file contained biogenic emission diagnostic output (B3GTS).

In total, we conducted four annual CMAQ simulations for 2016: one with unaltered emission data (simulation orig) and three with altered precision emission data by setting *n* to five (A05), four (A04), and three (A03) for all emission input files (gridded_no_rwc, gridded_rwc, ptnonipm, ptegu, ptagfire, ptfire, ptfire_othna, pt_oilgas, cmv_c3_12, cmv_c1c2_12, and othpt) utilized by CMAQ for this study. On the output side, direct CMAQ outputs (ACONC, APMDIAG, DRYDEP, and WETDEP1) from the A05, A04, and A03 (in which A0n signifies an altered simulation which utilized altered precision emission data to *n* significant digits) simulations were similarly altered to possess five, four, or three significant digits (denoted as FX05, FX04, and FX03, respectively, in which FX0n signifies an altered precision case which was post-processed by an A0n simulation’s CMAQ output). Emission input and CMAQ output data were then compressed separately by gzip (GNU Gzip, https://www.gnu.org/software/gzip, last access: 11 April 2022) and bzip2 (https://www.sourceware.org/bzip2, last access: 11 April 2022) for all simulations and cases to determine compression efficiency in terms of the reduction of disk space. In summary, there are four separate simulations (called orig or abbreviated as A0n) and nine additional, altered precision output cases (abbreviated as FX0n). For example, a CMAQ simulation that was run with emission data that were processed with *n* equals five significant digits, then post-processed to possess three significant digits, is denoted as A05FX03 (see Table 2 for a full list of simulations and cases).

Simulated numerical, or predictive, accuracy was analyzed against concentrations of particulate matter with diameter less than 2.5 μm (PM_2.5_), ozone (O_3_), ammonia (NH_3_), the wet-deposition rates of sodium (Na), ammonium (NH_4_), chlorine (Cl), nitrate (NO_3_), sulfate (SO_4_), and the dry-deposition rate of O_3_ for all simulations and cases. PM_2.5_ and O_3_ were evaluated at in situ stations from the dataset of the United States Environmental Protection Agency’s (US EPA) Air Quality System (AQS; Fig. 1b). Ammonia (NH_3_) was evaluated at in situ stations utilizing observations from the Ammonia Monitoring Network (AMON; Fig. 1c). Hourly observations of O_3_ were processed to calculate the maximum 8 h daily average concentrations (MDA8) and paired in space and time with calculated MDA8 O_3_ from post-processed CMAQ output. Likewise, daily averaged PM_2.5_ observations and 2-week-averaged NH3 observations were used to evaluate CMAQ. Observed values are paired with the volume-averaged pollutant estimate from CMAQ’s surface layer’s grid cell containing the air quality monitoring site (i.e., nearest neighbor). Statistical metrics were also calculated by pairing gridded values from the orig simulation (considered observed values) and the altered precision simulations and cases (considered the predicted values). Tabulated statistical metrics for grid–grid pairing was computed by taking the mean of hourly, statistical metrics.

Typical statistical metrics including mean bias (MB), correlation coefficient (*r*), root mean square error (RMSE), and normalized mean bias (NMB) are used to evaluate all chemical species in this analysis at different temporal intervals and for different pairing methodologies (either grid–point or grid–grid) which includes regional stratification (based on regions from Fig. 1a) for several figures. The utilized statistical metrics are denoted below in Eq. (1) through Eq. (4):

(1) $$\mathrm{MB}=\frac{1}{N}\cdot \overset{N}{\underset{i=1}{\Sigma }}\left(Y_{i}-X_{i}\right),$$

(2) $$r=\frac{1}{N-1}\cdot \frac{\overset{N}{\underset{i=1}{\Sigma }}\left(\left(X_{i}-\bar{X}\right)\cdot \left(Y_{i}-\bar{Y}\right)\right)}{\sigma_{X}\cdot \sigma_{Y}},$$

(3) $$\text{RMSE}=\sqrt{\frac{\overset{N}{\underset{i=1}{\Sigma }}{\left(Y_{i}-X_{i}\right)}^{2}}{N}},$$

(4) $$\mathrm{NMB}=\frac{\overset{N}{\underset{i=1}{\Sigma }}\left(Y_{i}-X_{i}\right)}{\overset{N}{\underset{i=1}{\Sigma }}\left(X_{i}\right)}\cdot 100\text{\%},$$

where *N* is the total number of observed and predicted pairs, *X* is the observed value, *Y* is predicted value, σ is the sample standard deviation of a distribution, and the overbars in Eq. (2) refer to the sample mean of a distribution. Although many compression toolsets exist and optimization is dependent on multiple factors (Kryukov et al., 2020), gzip and bzip2 are the most public, reliable, and widely used compressors. Both utilities are lossless compression algorithms which are available for Linux users. In terms of functionality, gzip uses a compression algorithm called Deflate (Deutsch, 1996) which reduces sequences of datasets by incorporating a combination of LZ77 dictionary coding (Ziv and Lempel, 1977) and Huffman entropy coding (Huffman, 1952). In comparison, bzip2 uses the Burrows–Wheeler (Burrows and Wheeler, 1994) algorithm which sorts all possible rotations of an input lexically and forms an output by concatenating the last character from the sorted list. In terms of compression ratio, bzip2 is notably better than gzip, however, with respect to compression speed, gzip is significantly faster than bzip2. Due to their availability and efficiency, both gzip and bzip2 are utilized in this study (default settings).

## 3 Results

### 3.1 Data storage

**Figure 2.**
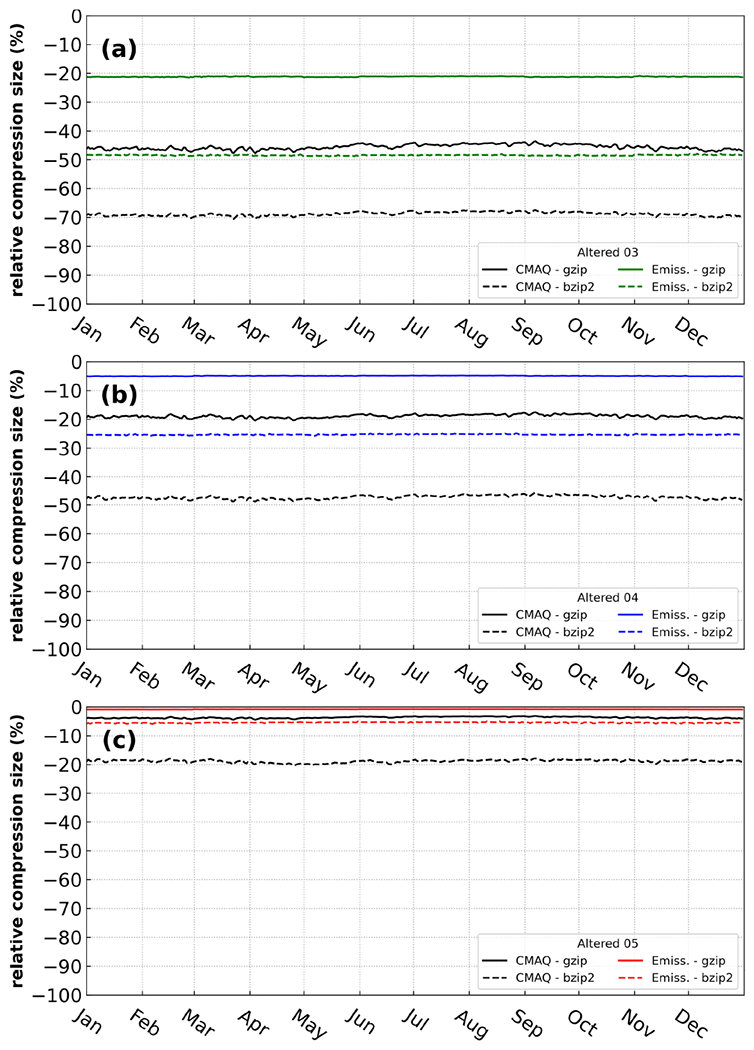
Relative compression size of two utilities, gzip (solid line) and bzip2 (dotted line), on daily emission files (labeled as Emiss.) and direct CMAQ output (labeled as CMAQ) for 2016 with reduced precision settings: 5, 4, and 3 (labeled as Altered 05, Altered 04, and Altered 03, respectively). Negative values indicate better compression efficiency.

The CMAQ input and output data are stored for future analyses and to ensure the reproducibility of modeling studies which demands a tremendous amount of disk space for input and output files. Therefore, we propose easing the disk-space burden by utilizing efficient compression algorithms. For this section of the analysis, two popular, reliable, and efficient compression utilities, gzip and bzip2, were utilized to determine compression efficiency with respect to emission input (emissions mentioned in Sect. 2.) files and CMAQ output (mentioned in Sect. 2. including CGRID, CONC, and SOILOUT) files. Both compression utilities were applied daily to compress emission input and CMAQ output files throughout the entirety of the 2016 simulation (Fig. 2).

The gzip compression utility reduced the file sizes, on average by 1 %, 5 %, and 21 %. This translates into about 5, 26, and 111GB actual difference between the compressed orig case and the compressed A05, A04, and A03 emission datasets for the entire year of 2016, respectively. The reduction in file size (using gzip) was more substantial when applied to reduced precision CMAQ output, with an average reduction in file size of 4 %, 19 %, and 67 %. This means about 167, 839, and 2016GB actual difference between the orig case and FX05, FX04, and FX03, respectively for the entire year. With the bzip2 utility, the reduction in magnitude is much larger than with gzip, with an average reduction of file size equal to 6 %, 25 %, and 48% (actual differences are about 27, 126, and 241 GB, respectively for A05, A04, and A03 emission files and 19 %, 47 %, and 69% (actual differences are about 856, 2142, and 3115 GB, respectively) for the compressed CMAQ output. Thus, bzip2 is found to be a more effective tool than gzip by roughly 5 %, 20 %, and 27% for emission data and 15 %, 28% and 23% for CMAQ output, for reduced precision by keeping 5, 4, and 3 significant digits (reduced precision emissions and reduced precision output data), respectively.

### 3.2 Runtime

**Figure 3.**
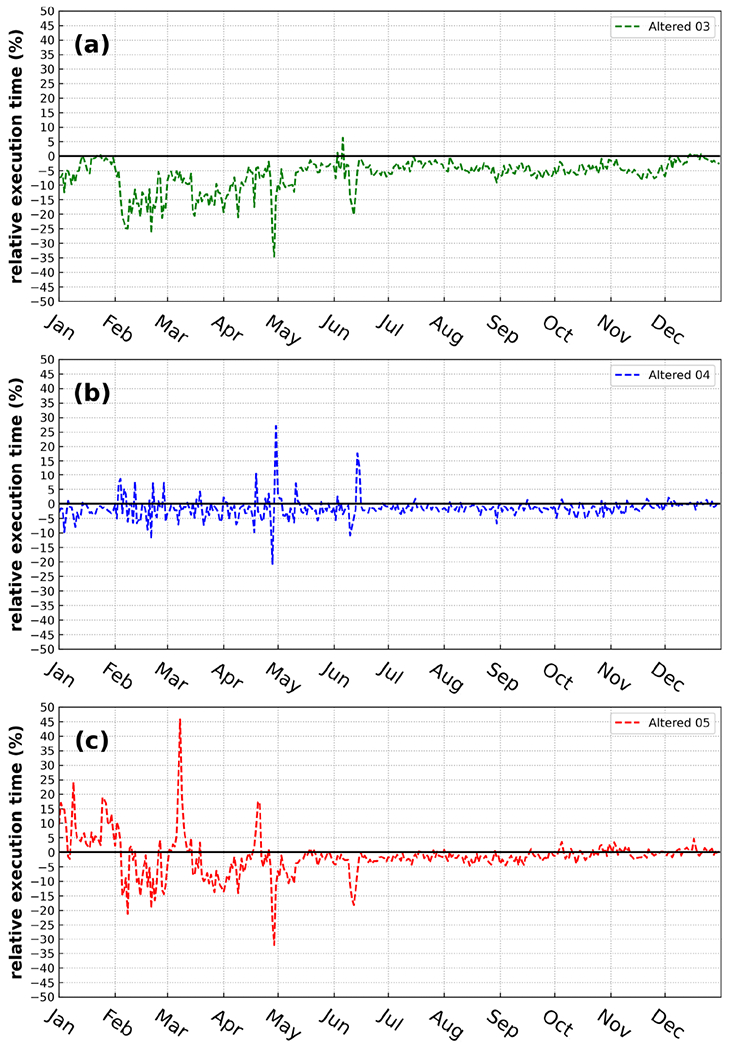
Relative daily runtime with respect to different adjusted emission input for the A03, A04, and A05 simulations for 2016.

We examined daily runtime (captured by an MPI function called MPI_WTIME) for CMAQ using emission data prepared with truncations of A05, A04, and A03 compared with running CMAQ with unaltered (*orig*) emission data (Fig. 3). Even though the simulations were not performed in a dedicated environment (results are not entirely consistent due to the allocation of resources when the simulations were initialized), the daily runtimes for A05, A04, and A03 were lower than the runtime of the orig simulation in most of the days. The total runtimes for the A05, A04, and A03 simulations were 3.13, 2.94, and 12.84 h faster than the orig case (2 %, 2 %, and 7 %, respectively of the relative reduction of runtime). There are two possible explanations for such behavior: first, during the execution of each case, CMAQ competed for I/O resources with other tasks on the system. As a result, an I/O bottleneck could explain spikes in relative runtime on certain simulation days (Fig. 3). Second, a change in emission input (due to the reduced precision emission data) could alter the pathway for the aerosol dynamics calculation.

### 3.3 Accuracy

**Figure 4.**
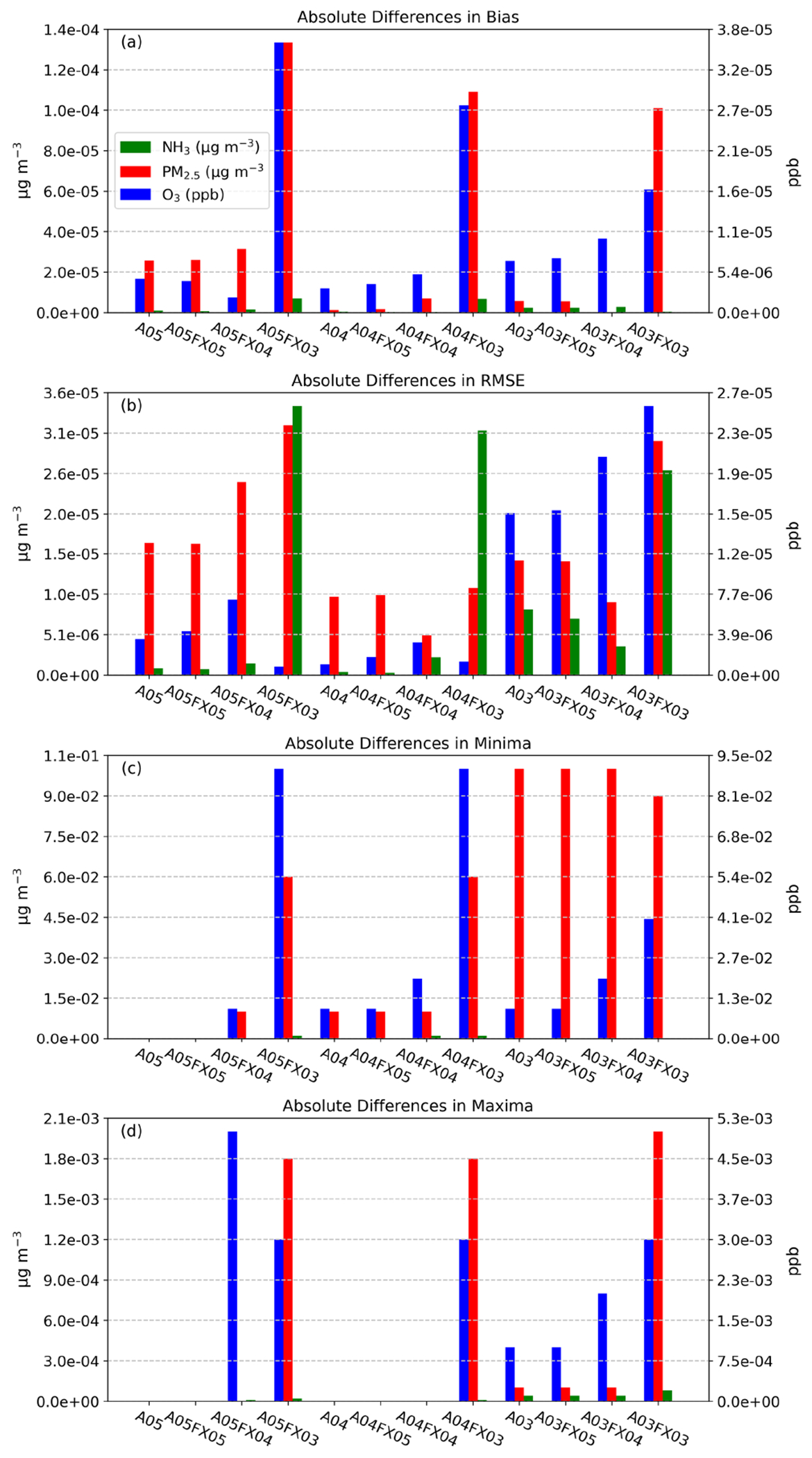
Absolute differences in bulk statistical metrics for daily PM_2.5_, MDA8 O_3_, and 2-week-averaged NH_3_ between the orig simulation and the altered simulations and cases. Bulk statistical metrics were calculated for all model–observation pairs at in situ stations.

The accuracy of each case is first examined grid-to-point between modeled output and in situ observations (Fig. 1; AQS and AMON) for all available model–measurement pairs throughout 2016. In general, to gauge the accuracy of CMAQ, bulk statistical metrics of bias, NMB, *r*, and RMSE have been provided in Table 3 for the orig simulation. To compare bulk statistical metrics to the orig simulation, the absolute difference in bias, RMSE, minima (minimum difference between all model and observation pairs), and maxima was calculated with respect to the altered simulations and cases for daily PM_2.5_, MDA8 O_3_, and 2-week-averaged NH_3_. Overall, negligible differences are apparent (Fig. 4). For example, the maximum absolute, bulk statistical difference between the orig simulation and the altered cases and simulations for daily PM_2.5_, MDA8 O_3_, and 2-week-averaged NH_3_ did not exceed 1.4 × 10^−4^, 3.6 × 10^−5^, 1.1 × 10^−1^, and 5.3 × 10^−3^ μg m^−3^ or ppb for bias, RMSE, minima, and maxima, respectively. Therefore, differences in terms of maximum absolute, bulk statistical differences are quite small amongst the unaltered simulation (orig) and the altered simulations and cases.

**Figure 5.**
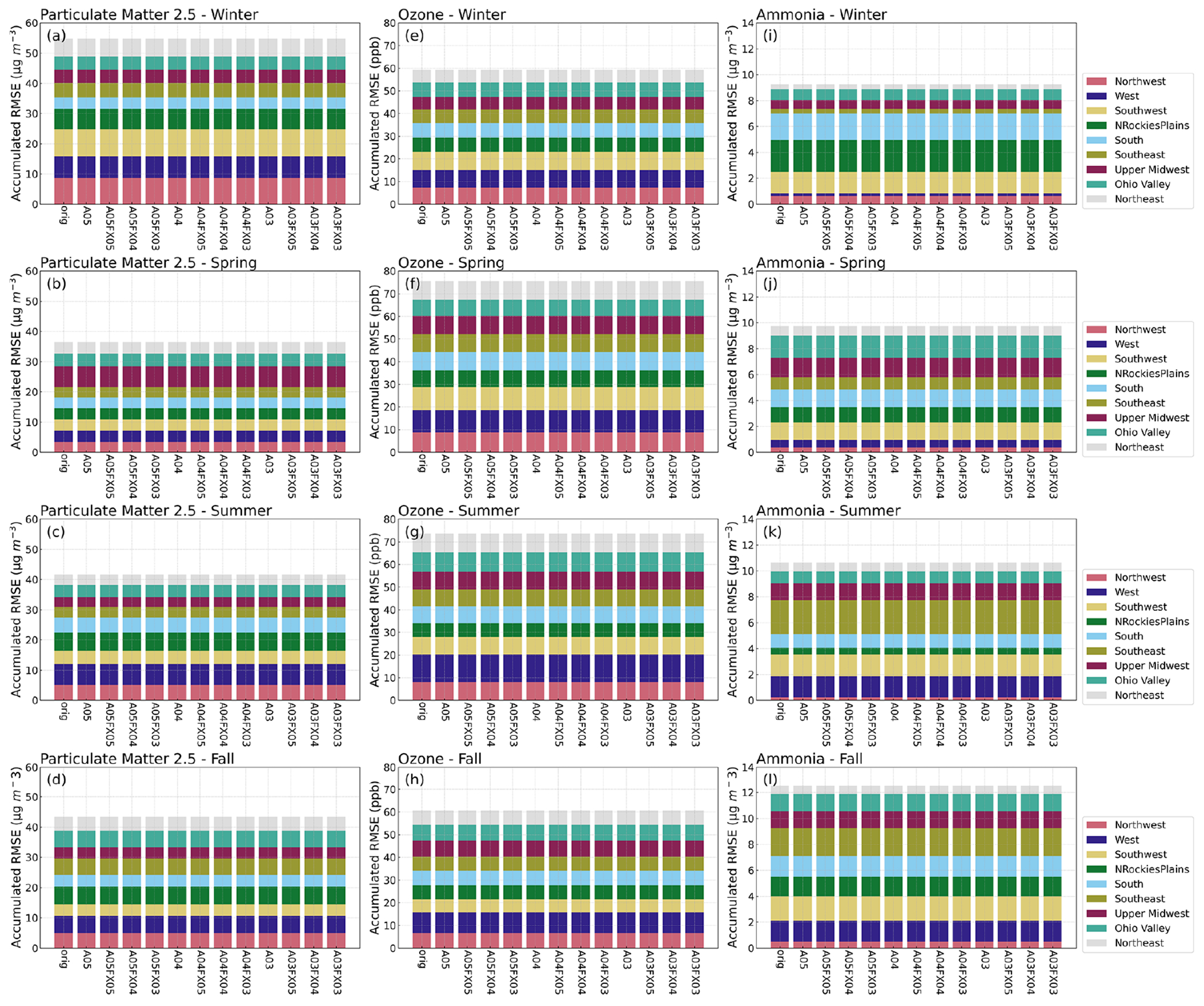
Stacked bar plots of RMSE (*y* axis) stratified by region (color), simulation and case (*x* axis), and season (subplot) for daily PM_2.5_, MDA8 O_3_, and 2-week-averaged NH_3_ calculated from in situ observation.

Bulk statistical results with respect to in situ observations and compared to the orig simulation (Fig. 4) are encouraging; differences are small, ignoring regional or temporal stratification. To determine if statistical results fluctuate spatially (by region) and or temporally (by season), RMSE was computed for nine different subregions (regions are portrayed in Fig. 1) across the United States for four seasons (winter, spring, summer, and fall) from the mentioned observation and model pairs. Each region’s RMSE was stacked together, by simulation and case, and plotted as “accumulated RMSE” by species. Likewise, results are negligible for daily PM_2.5_, MDA8 O_3_, and 2-week-averaged NH_3_, respectively (Fig. 5) for all regional and temporal stratifications and for all simulations and cases.

**Figure 6.**
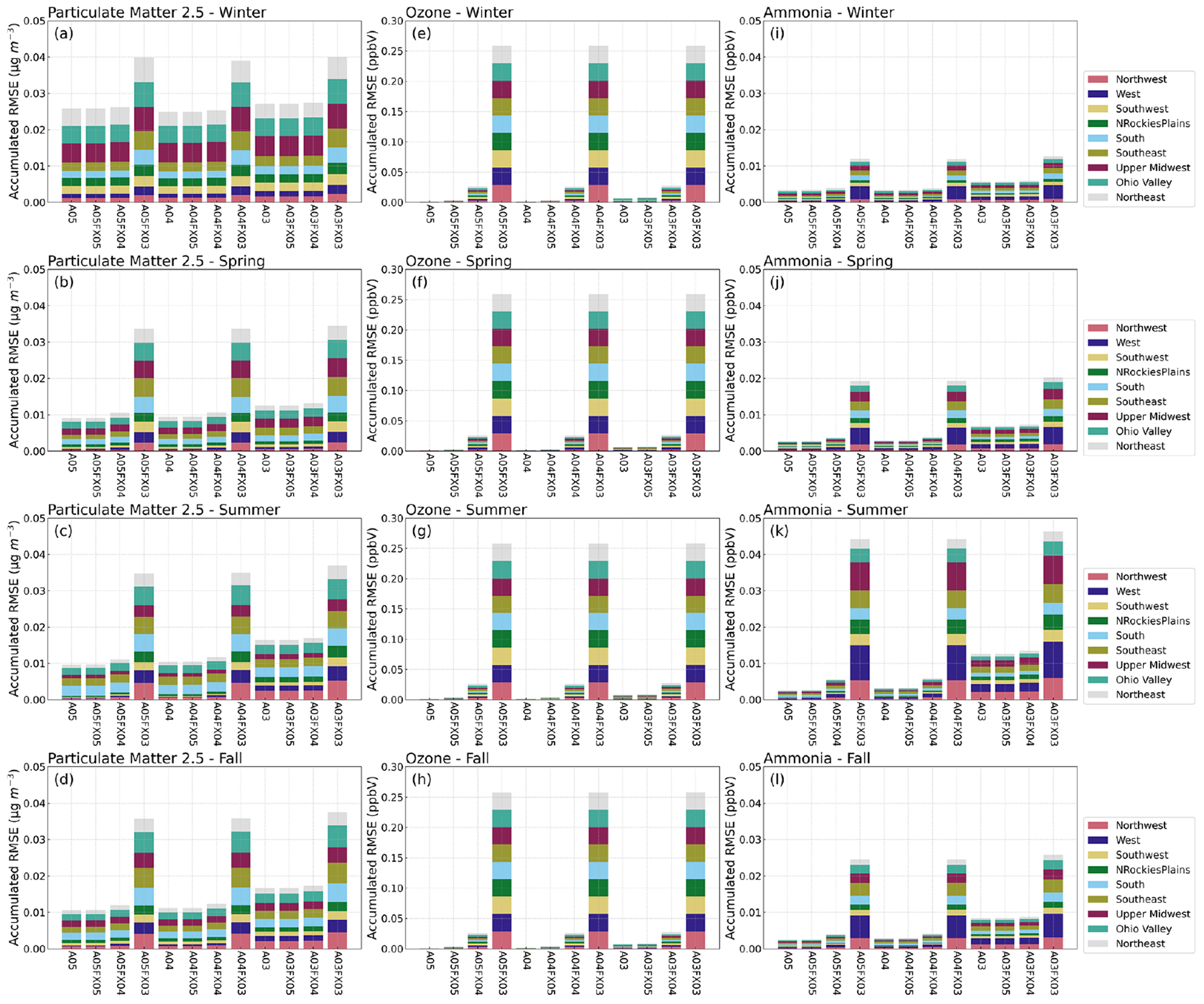
Stacked bar plots of changes to RMSE (*y* axis) stratified by region (color), simulation and case (*x* axis), and season (subplot) for hourly PM_2.5_, O_3_, and NH_3_ calculated from grid–grid pairs with respect to the orig simulation.

Results indicate that all simulations and cases have negligible differences in terms of bulk statistical metrics across the United States and considering regional and temporal stratifications. Statistical results conducted on in situ observations were redone (methodologically) at the grid level for hourly PM_2.5_, O_3_ and NH_3_, using the orig simulation (as the observed field) with respect to the altered precision simulations and cases (predicted fields). The RMSE was first calculated for all hourly grid–grid pairs for PM_2.5_, O_3_, and NH_3_. Only cells that are within each region (Fig. 1a), within the contiguous US, were used to calculate hourly RMSE for all available regional pairs. Next, the average, hourly RMSE was calculated for each season and region based on spatial and temporal masking using the regions portrayed in Fig. 1a. All stratifications were grouped together as accumulative, stacked bar plots for different seasons by simulation or case. Although differences are evident (Fig. 6), the scale of such differences is quite small. For example, the total accumulative RMSE for PM_2.5_, O_3_, and NH_3_ (sum of all region’s RMSE) did not exceed 0.04 μg m^−3^, 0.3 ppbV, and 0.05 ppbV, respectively for all cases and for all seasons.

**Figure 7.**
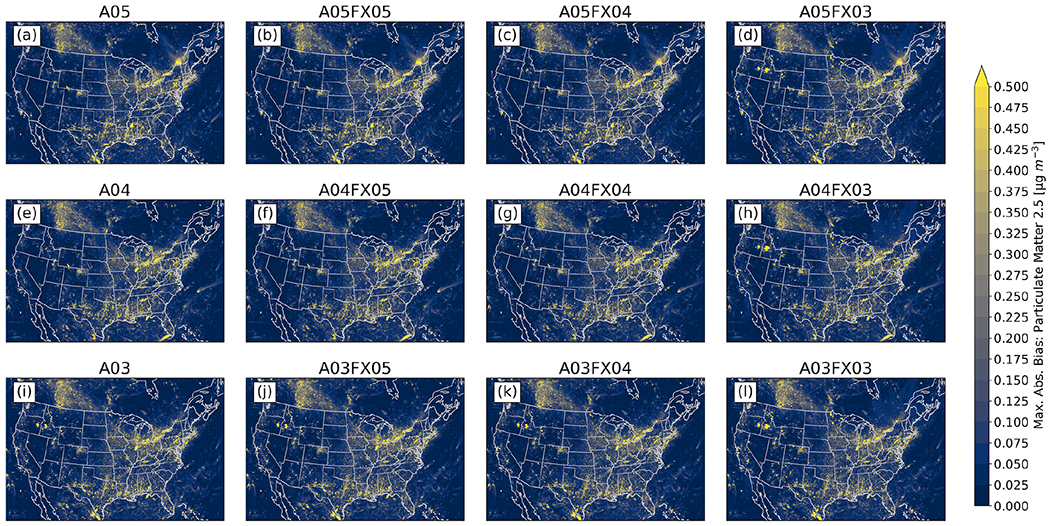
Maximum absolute bias (versus the orig simulation) for PM_2.5_ calculated from hourly output for all simulations and cases.

**Figure 8.**
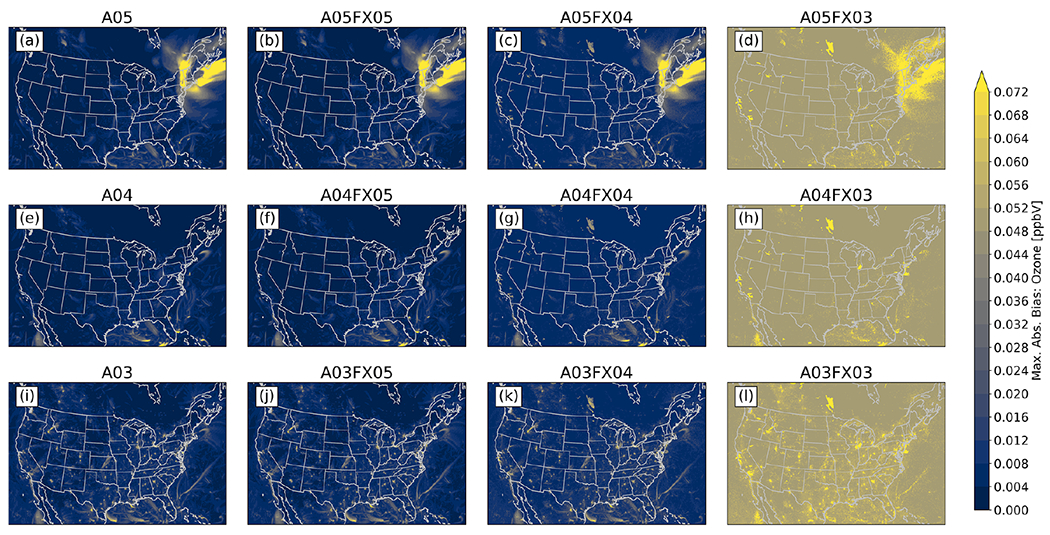
Maximum absolute bias (versus the orig simulation) for O_3_ calculated from hourly output for all simulations and cases.

**Figure 9.**
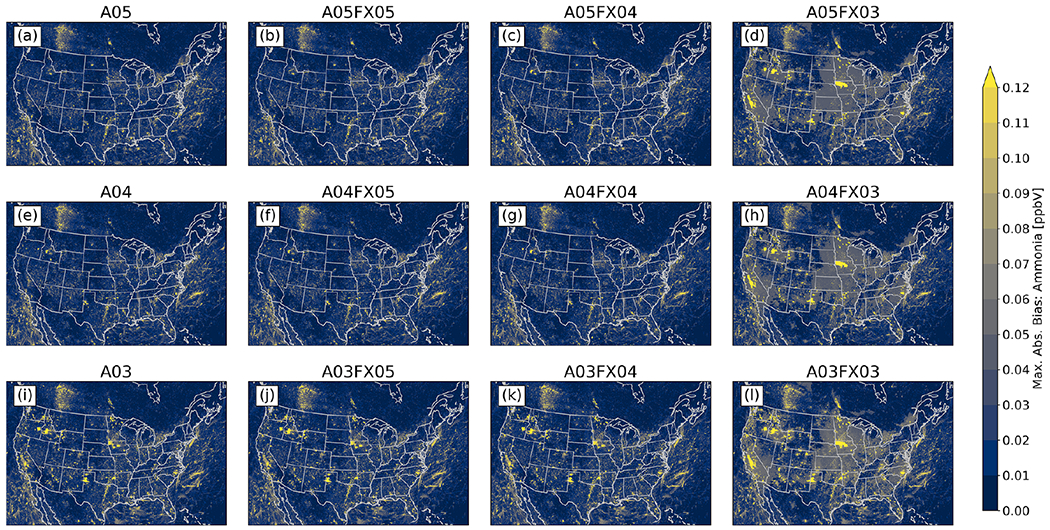
Maximum absolute bias (versus the orig simulation) for NH_3_ calculated from hourly output for all simulations and cases.

Additionally, the maximum absolute bias for all grid cells was determined spatially between the orig simulation and the altered simulations and cases throughout 2016 for PM_2.5_, O_3_, and NH_3_ from gridded, hourly (CMAQ) output. For PM_2.5_, all simulations and cases performed similarly, in which no visual differences are apparent (Fig. 7). For O_3_ (Fig. 8) and NH_3_ (Fig. 9), however, the differences become relatively large for cases *n* = 3. In fact, for both species, spatial and magnitude error visibly increase with fewer significant digits (simulations and cases). For example, the maximum absolute bias is largest for the A03 simulations and even worse for the FX03 altered precision cases, ignoring the artifact of error across the Northeastern United States for O_3_ for the A05 simulations and cases (induced by the A05 simulation). The maximum absolute bias ranges, found by taking the range of all altered precision cases, for PM_2.5_, O_3_, and NH_3_ are 46.77 μg m^−3^, 0.4265 ppbV, and 18.78 ppbV, respectively (Table 4). The minimum absolute bias ranges for PM_2.5_, O_3_, and NH_3_ are 5.573 μg m^−3^, 0.5091 ppbV, 9.778 ppbV (Table 4), respectively. Based on range, error can potentially be quite large compared to the statistics provided in Fig. 6, however, large-scale error is not persistent based on the small accumulated RMSE for all regions grouped by CMAQ simulation and case (Fig. 6). For example, for PM_2.5_, the maximum positive bias was (roughly) between 41 and 51 μg m^−3^ for the FX03 cases (Table 4). Upon further investigation, this relatively significant error occurred at one grid cell because of an anomalous wildfire (Pioneer wildfire in Idaho from July to September of 2016). Prior to the onset of the Pioneer wildfire and after the wildfire was extinguished, PM_2.5_ returned to normal levels with respect to the orig simulation for FX03 cases. Regardless, total accumulated values did not exceed 0.04 μg m^−3^, 0.3 ppbV, and 0.05 ppbV for PM_2.5_, O_3_, and NH_3_ respectively. Since errors associated with Figs. 7–9 are predominately small (maximum absolute bias), relatively large error (similar to the discrepancies in bias for PM_2.5_ for the FX03 cases) is associated with brief spikes of certain species within and around source regions.

**Figure 10.**
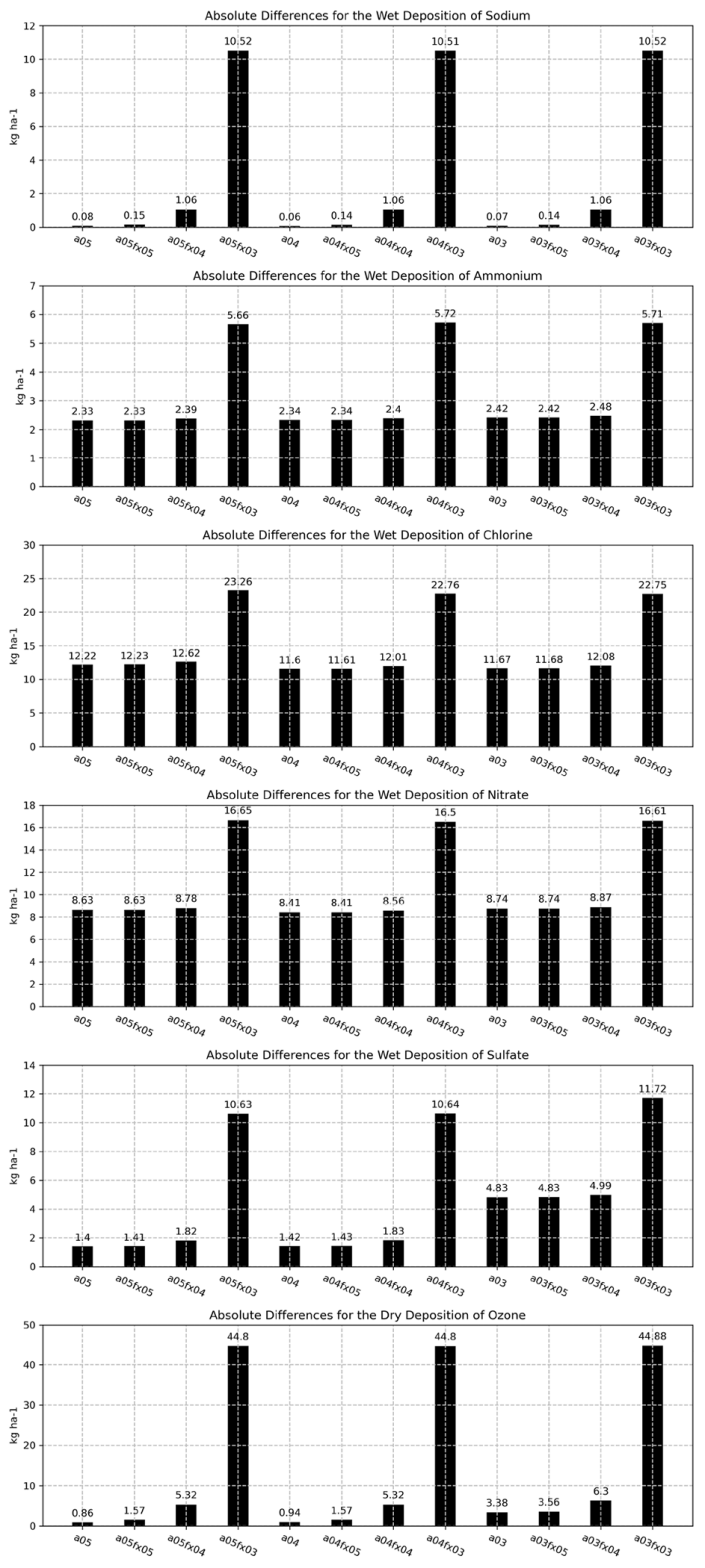
Total absolute bias difference between the orig simulation and the altered cases and simulations by deposition rate (row) throughout 2016 utilizing hourly output.

The final aspect of this evaluation explores differences of important deposition rates using bar plots which depict the sum of hourly absolute differences (for all cells across the domain) between the orig simulation and the altered simulations and cases. Bar plots were created for the wet-deposition rates of sodium (Na), ammonium (NH_4_), chlorine (Cl), nitrate (NO_3_), sulfate (SO_4_), and the dry-deposition rate of O_3_ for all altered precision simulations and cases. For all deposition rates, all 3 cases, A05FX03, A04FX03, and A03FX03, performed equally poor, relatively speaking, with respect to the orig simulation. The A05FX04, A04FX04, and A03FX04 cases performed nearly identically to the A05FX05, A04FX05, and A03FX05 cases for all deposition rates, excluding the wet-deposition rate of sodium and sulfate and the dry-deposition rate of ozone. The altered precision 5 cases ( A05FX05, A04FX05, and A03FX05) and the altered simulations (A05, A04, and A03) performed nearly identically to the orig simulation for all deposition rates. Overall, considering that each bar plot in Fig. 10 represents the sum of all hourly differences across the entire domain, all species, simulations, and cases performed similarly with respect to the orig case, and hence, amongst each other. For comparison purposes, the annual sum, considering all grid cells within the contiguous United States, for the wet-deposition rates of sodium, ammonium, chlorine, nitrate, and sulfate are 1.42 × 10^5^, 6.69 × 10^4^, 21.75 × 10^4^, 2.58 × 10^5^, and 1.72 × 10^5^ kg ha^−1^, respectively for the base simulation (orig). Similarly, the annual sum for the dry-deposition rate of ozone (contiguous United States) is 2.78 × 10^6^ kg ha^−1^ for the base simulation.

No error accumulation due to the non-systematic changes in model inputs (changing precision introduces both positive and negative changes in a spatially and temporally random manner) can occur over the course of the annual simulation for chemical species of interest such as O_3_ and PM_2.5_. Their lifetimes are much shorter than a year, i.e., their simulated budgets within the continental-scale modeling domain are repeatedly exchanged through transport, emissions, and chemical and physical sinks. All simulations (orig, A05, A04, and A03) are numerically stable (no compounding error over time).

## 4 Conclusions

We have demonstrated that altering data by keeping a specified number of significant digits in terms of emission input and/or simulated output, increased compression efficiency based on two different, popular compression utilities (gzip and bzip2). For emission data, bzip2 performed far better than gzip and provided compression reduction, on average, by 6 %, 25 %, and 48 %, and 19 %, 47 %, and 69 % for output data for the A05, A04, and A03 cases, respectively, compared to the orig case. In terms of daily simulation runtime for the entire simulation year, the A05, A04, and A03 simulations were faster than the orig simulation in an undedicated HPC system for most simulation days.

As for accuracy, results for all studied simulations, either with altered precision emission only, or with altered precision emission plus altered precision output, produced numerically insignificant differences. For example, the maximum absolute, bulk statistical difference between the orig simulation and the altered cases and simulations for daily PM_2.5_, MDA8 O_3_, and 2-week-averaged NH_3_ did not exceed 1.4 × 10^−4^, 3.6 × 10^−5^, 1.1 × 10^−1^, and 5.3 × 10^−3^ μg m^−3^ or ppb for bias, RMSE, minima, and maxima, respectively. Similarly, a small range in values is replicated for all other bulk statistical metrics such as MB, *r*, and RMSE. Results stratified by region and season are similar to those for bulk statistics. Based on the in situ evaluation, simulation performance is very similar amongst all cases, with visible differences for the A03 simulation and the FX03 cases in which error is spatially detected in Figs. 7–9.

Statistical inconsistencies arise when comparing grid–grid values of hourly PM_2.5_, O_3_, and NH_3_ versus the orig simulation. Results indicate that similarities amongst the orig simulation decreases with fewer significant digit simulations and cases when analyzing the stacked and stratified (region and season) RMSE bar plot (Fig. 6). More specifically, performance with respect to the orig simulation is worse for the A03 simulation and for the FX03 cases as well. Such discrepancies do not occur consistently based on results provided by bar plots of statistical metrics of deposition rates (Fig. 10). Instead, errors appear to be confined to source regions at specific instances based on the maximum absolute (hourly) error spatial plots with respect to the orig simulation (Figs. 7–9).

In summary, altering datasets by truncation to retain fewer significant digits significantly improved data compression and slightly improved runtime. Based on the thorough, yet spatially limited, in situ evaluation, this study has shown this proposed technique did not compromise model accuracy based on an evaluation of simulations and cases at in situ locations compared to current air quality thresholds for daily PM_2.5_, MDA8 O_3_, and 2-week-averaged NH_3_. These results show the optimal benefit of altering CMAQ input data by keeping three significant digits, then subsequently keeping four significant digits for CMAQ output data. In addition, this proposed technique could be beneficial for groups that perform complex air quality modeling and want to improve disk-space management while negligibly impacting the accuracy of the simulations. Based on the success of this study, we propose testing these techniques on the rest of CMAQ input files such as initial conditions, boundary conditions, and meteorological data to determine the viability of these techniques to more adeptly manage disk space without compromising the quality of the CMAQ simulations used for research and to develop air quality management strategies.

## Figures and Tables

**Table 1.** Examples of precision-reducing transformations of floating points from their original forms (first column) to their altered precision forms (second to fourth column).

Original (orig)	Altered 5 (A05)	Altered 4 (A04)	Altered 3 (A03)
0.005666635	0.0056666	0.005667	0.00567
3.437405 × 10^−6^	3.4374 × 10^−6^	3.437 × 10^−6^	3.44 × 10^−6^
0.0005319762	0.00053198	0.000532	0.000532
3.437 × 10^−6^	3.437 × 10^−6^	3.437 × 10^−6^	3.44 × 10^−6^
100 150.0	100 150.0	100 200.0	100 000.0

**Table 2.** Setup of all simulations (orig, A05, A04, and A03) and cases analyzed in this study.

Unaltered emission data		Altered precision emission data					
(a)	Simulation: orig	(b)	Simulation: A05	(c)	Simulation: A04	(d)	Simulation: A03
		Altered precision CMAQ output					
		(e)	Case: A05FX05	(h)	Case: A04FX05	(k)	Case: A03FX05
		(f)	Case: A05FX04	(i)	Case: A04FX04	(l)	Case: A03FX04
		(g)	Case: A05FX03	(j)	Case: A04FX03	(m)	Case: A03FX03

**Table 3.** Annual bulk statistical metrics for all grid–point pairs for the unaltered simulation (orig) binned by species (row) and statistic (column).

Case	Bias	NMB (%)	*r*	RMSE
PM_2.5_ (μg m^−3^)	−0.02828948	−0.37369379	0.53041275	5.01579136
MAD8 O_3_ (ppb)	−1.70888518	−4.07590175	0.76393761	7.93497772
NH_3_ (μg m^−3^)	−0.42796669	−35.05920328	0.51400293	1.28576807

**Table 4.** Maximum and minimum biases (altered – orig) calculated from hourly CMAQ output for all simulations and cases with respect to the orig simulation across all grid cells.

Case	PM_2.5_ (μg m^−3^)		Ozone (ppbV)		Ammonia (ppbV)	

	Max.	Min.	Max.	Min.	Max.	Min.
A05FX05	4.40819836	−4.69252777	0.260878	−0.08337	0.893507	−0.61453
A05FX04	4.40777397	−4.69240379	0.263882	−0.08437	0.893806	−4.01074
A05FX03	51.17382812	−9.62011719	0.499962	−0.50085	19.64355	−4.86621
A05	4.40821075	−4.69258881	0.260483	−0.08343	0.893517	−0.61455
A04FX05	4.99303246	−4.70221233	0.136284	−0.16548	0.875244	−1.14815
A04FX04	4.99263382	−4.70223236	0.136154	−0.16448	1.275146	−4.01074
A04FX03	51.1640625	−9.51953125	0.503494	−0.50282	19.64355	−5.02832
A04	4.99302673	−4.70224953	0.13604	−0.16512	0.867432	−1.14854
A03FX05	11.09228516	−6.66992188	0.223785	−0.22272	4.146118	−7.44141
A03FX04	11.54589844	−10.265625	0.225784	−0.22272	4.446045	−7.0415
A03FX03	41.18359375	−9.46972656	0.562561	−0.59249	19.64355	−10.3923
A03	11.17675781	−7.01953125	0.224041	−0.22235	4.187866	−7.47461
